# Treatment of severely ill COVID-19 patients with anti-interleukin drugs (COV-AID): A structured summary of a study protocol for a randomised controlled trial

**DOI:** 10.1186/s13063-020-04453-5

**Published:** 2020-06-03

**Authors:** Bastiaan Maes, Cedric Bosteels, Elisabeth De Leeuw, Jozefien Declercq, Karel Van Damme, Anja Delporte, Bénédicte Demeyere, Stéfanie Vermeersch, Marnik Vuylsteke, Joren Willaert, Laura Bollé, Yuri Vanbiervliet, Jana Decuypere, Frederick Libeer, Stefaan Vandecasteele, Isabelle Peene, Bart Lambrecht

**Affiliations:** VIB-UGent Inflammatie-researchcentrum, Oost-Vlaanderen, Ghent, Belgium

**Keywords:** COVID-19, Randomised controlled trial, protocol, systemic cytokine release syndrome, hypoxic respiratory failure, interleukin 6 blockade, interleukin 1 blockade, Anakinra, Siltuximab, Tocilizumab, Acute Respiratory Distress Syndrome

## Abstract

**Objectives:**

The purpose of this study is to test the safety and effectiveness of individually or simultaneously blocking IL-6, IL-6 receptor and IL-1 versus standard of care on blood oxygenation and systemic cytokine release syndrome in patients with COVID-19 coronavirus infection and acute hypoxic respiratory failure and systemic cytokine release syndrome.

**Trial design:**

A phase 3 prospective, multi-center, interventional, open label, 6-arm 2x2 factorial design study.

**Participants:**

Subjects will be recruited at the specialized COVID-19 wards and/or ICUs at 16 Belgian participating hospitals. Only adult (≥18y old) patients will be recruited with recent (≤16 days) COVID-19 infection and acute hypoxia (defined as PaO2/FiO2 below 350mmHg or PaO2/FiO2 below 280 on supplemental oxygen and immediately requiring high flow oxygen device or mechanical ventilation) and signs of systemic cytokine release syndrome characterized by high serum ferritin, or high D-dimers, or high LDH or deep lymphopenia or a combination of those, who have not been on mechanical ventilation for more than 24 hours before randomisation. Patients should have had a chest X-ray and/or CT scan showing bilateral infiltrates within the last 2 days before randomisation. Patients with active bacterial or fungal infection will be excluded.

**Intervention and comparator:**

Patients will be randomized to 1 of 5 experimental arms versus usual care. The experimental arms consist of Anakinra alone (anti-IL-1 binding the IL-1 receptor), Siltuximab alone (anti-IL-6 chimeric antibody), a combination of Siltuximab and Anakinra, Tocilizumab alone (humanised anti-IL-6 receptor antibody) or a combination of Anakinra with Tocilizumab in addition to standard care. Patients treated with Anakinra will receive a daily subcutaneous injection of 100mg for a maximum of 28 days or until hospital discharge, whichever comes first. Siltuximab (11mg/kg) or Tocilizumab (8mg/kg, with a maximum dose of 800mg) are administered as a single intravenous injection immediately after randomization.

**Main outcomes:**

The primary end point is the time to clinical improvement defined as the time from randomization to either an improvement of two points on a six-category ordinal scale measured daily till day 28 or discharge from the hospital or death. This ordinal scale is composed of (1) Death; (2) Hospitalized, on invasive mechanical ventilation or ECMO; (3) Hospitalized, on non-invasive ventilation or high flow oxygen devices; (4) Hospitalized, requiring supplemental oxygen; (5) Hospitalized, not requiring supplemental oxygen; (6) Not hospitalized.

**Randomisation:**

Patients will be randomized using an Interactive Web Response System (REDCap). A 2x2 factorial design was selected with a 2:1 randomization regarding the IL-1 blockade (Anakinra) and a 1:2 randomization regarding the IL-6 blockade (Siltuximab and Tocilizumab).

**Blinding (masking):**

In this open-label trial neither participants, caregivers, nor those assessing the outcomes are blinded to group assignment.

**Numbers to be randomised (sample size):**

A total of 342 participants will be enrolled: 76 patients will receive usual care, 76 patients will receive Siltuximab alone, 76 patients will receive Tocilizumab alone, 38 will receive Anakinra alone, 38 patients will receive Anakinra and Siltuximab and 38 patients will receive Anakinra and Tocilizumab.

**Trial Status:**

COV-AID protocol version 3.0 (15 Apr 2020). Participant recruitment is ongoing and started on April 4^th^ 2020. Given the current decline of the COVID-19 pandemic in Belgium, it is difficult to anticipate the rate of participant recruitment.

**Trial registration:**

The trial was registered on Clinical Trials.gov on April 1st, 2020 (ClinicalTrials.gov Identifier: NCT04330638) and on EudraCT on April 3rd 2020 (Identifier: 2020-001500-41).

**Full protocol:**

The full protocol is attached as an additional file, accessible from the Trials website (Additional file 1). In the interest in expediting dissemination of this material, the familiar formatting has been eliminated; this Letter serves as a summary of the key elements of the full protocol.

## Supplementary information

**Additional file 1.** Full study protocol.

## Data Availability

Not applicable.

