# Correction to: Treatment of severely ill COVID-19 patients with anti-interleukin drugs (COV-AID): A structured summary of a study protocol for a randomised controlled trial

**DOI:** 10.1186/s13063-020-04519-4

**Published:** 2020-06-22

**Authors:** Bastiaan Maes, Cedric Bosteels, Elisabeth De Leeuw, Jozefien Declercq, Karel Van Damme, Anja Delporte, Bénédicte Demeyere, Stéfanie Vermeersch, Marnik Vuylsteke, Joren Willaert, Laura Bollé, Yuri Vanbiervliet, Jana Decuypere, Frederick Libeer, Stefaan Vandecasteele, Isabelle Peene, Bart N. Lambrecht

**Affiliations:** VIB-UGent Inflammatie-researchcentrum, Oost-Vlaanderen, Ghent, Belgium

**Correction to: Trials (2020) 21:468**

**https://doi.org/10.1186/s13063-020-04453-5**

Following publication of the original article [1], we have been notified that an initial was missed from one of the author names.

Originally published author names:

- Bart Lambrecht

Correct author names:

- Bart N. Lambrecht

The original article has been corrected.
