# Supplementary material for: Treatment of severely ill COVID-19 patients with anti-interleukin drugs (COV-AID): A structured summary of a study protocol for a randomised controlled trial
Source: Trials. 2020 Jun 3;21:468. doi: 10.1186/s13063-020-04453-5 (PMC7267751; doi:10.1186/s13063-020-04453-5)
Supplement: Supplementary file 1 — Additional file 1. Full study protocol. [file 13063_2020_4453_MOESM1_ESM.pdf]

A prospective, randomized, factorial design, interventional study to compare the safety and efficacy of combinations of blockade of interleukin-6 pathway and interleukin-1 pathway to best standard of care in improving oxygenation and short- and long-term outcome of COVID-19 patients with acute hypoxic respiratory failure and systemic cytokine release syndrome.

Acronym / Protocol code	COV-AID: Treatment of COVID-19 patients with anti-interleukin drugs
Protocol version and date	Version 3.0 15/04/2020
Phase	Phase III
EudraCT n°	2020-001500-41
Sponsor	University Hospital Ghent,<br>C. Heymanslaan 10<br>9000 Ghent<br>Belgium
Financial/Material Support:	Belgian healthcare knowledge centre (KCE) Brussels
Coordinating Investigator:	**UZ Gent**<br>**Bart N. Lambrecht, MD, PhD**<br>Department of Internal Medicine & Pediatrics,<br>Department of Respiratory Medicine<br>University Hospital Ghent<br>Corneel Heymanslaan 10<br>9000 Ghent, Belgium<br>+32/93329110
Co-investigators:	**Multicentric trial in Belgium**

Gauthier Bouche, Catherine Legrand, Marc Buyse, Jillian Harrison, Nelle Stocquart, Hilde Nevens, Catherine Van Der Straeten and Frank Hulstaert contributed significantly.

A prospective, randomized, factorial design, interventional study to compare the safety and efficacy of combinations of blockade of interleukin-6 pathway and interleukin-1 pathway to best standard of care in improving oxygenation and short- and long-term outcome of COVID-19 patients with acute hypoxic respiratory failure and systemic cytokine release syndrome.

Protocol Co-ordinating Investigator signature page

I certify that I will conduct the study in compliance with the protocol, any amendments, GCP and the declaration of Helsinki, and all applicable regulatory requirements.

Investigator:

Name: Prof. Dr. B. Lambrecht
Function: Pulmonologist
Institution: UZ Ghent

Date: **Signature:**

A prospective, randomized, factorial design, interventional study to compare the safety and efficacy of combinations of blockade of interleukin-6 pathway, and interleukin-1 pathway to best standard of care in improving oxygenation and short- and long-term outcome of COVID-19 patients with acute hypoxic respiratory failure and systemic cytokine release syndrome.

Protocol Site Principal Investigator signature page

I certify that I will conduct the study in compliance with the protocol, any amendments, GCP and the declaration of Helsinki, and all applicable regulatory requirements.

Investigator:

Name:
Function:
Institution:

Date: **Signature:**

Protocol Amendment History:

Version Number	Date	Description of amendment
1.2	06APR2020	**section 7.1.3 p 27 SYLVANT ®,**<br>Will be given via single IV infusion at a dose of 11 mg / kg given over 1 hour as an intravenous infusion in glucose 50% → 5% due to typo error
2.0	06APR2020	Section 5.1 p21:<br>Recent (≤14 days of) of flu-like symptoms or malaise prior to randomization) infection with COVID-19 →<br>changed to ≤16 days
		section 5.2 p23: **frailty score**<br>exclusion criteria: clinical frailty score >2 → changed to clinical frailty score > 3<br>Section 5.1 p21, inclusion criteria: clinical frailty score deleted
		section 5.1 p21: **COVID-19 diagnosis:**<br>serology and emerging technologies added as diagnostic test
		Section 5.1 p21: **COVID-19 diagnosis**<br>Probable COVID-19 infection defined by chest CT-scan and clinical criteria added
2.1	10APR2020	Section 8.2 p28-29: **EDTA**<br>Two → four tubes of EDTA tube (10 ml)<br>Error, correct in schematic overview and sampling
3.0	15APR2020	Section 5.1 p21: IC1<br>Confident COVID diagnosis …
		Section 5.1:p 21 IC 4 clarification FiO_2
		Section 5.1: p21 added extra IC<br>Female subject need to use adequate contraception during treatment and 3 months after treatment
		Section 7.1.3 p26: Roactemra sc to IV clarification
		Section 8.4 p31: schematic overview<br>Procalcitonin explicit added in overview
		Section 12.6 p41 data safety monitoring board will be foreseen
		Section 7.1.3 p26 Dose justification added

TABLE OF CONTENTS

LIST OF ABBREVIATIONS 8
1. Protocol Summary 9
2. Rationale and background 11
2.1. Rationale 11
2.2. Background 13
2.3. Risk/Benefit Assessment 14
2.4. Primary Objectives 16
2.5. Secondary Objectives 16
3. End Points + Time Points 17
3.1. Primary End Points + Time Points 17
3.2. Secondary End Points + Time Points 17
4. Study design 19
4.1. Description of study design 19
4.2. End of Study Definition 20
4.2.1. For an individual subject 20
4.2.2. For the whole study 20
4.3. Estimated duration of the study 21
5. Inclusion and Exclusion Criteria 21
5.1. Inclusion Criteria 21
5.2. Exclusion Criteria 22
5.2.1. Screen failures 22
6. Target Population 23
6.1. Subjects 23
6.1.1. Number of subjects and planned recruitment rate 23
6.1.2. Withdrawal and replacement of subjects 23
6.2. Method of recruitment 24
6.3. Screening 24
7. Investigational Medicinal Product (IMP) 25
7.1. Name of the IMP 25
7.1.1. Composition and active substance of the IMP 25
7.1.2. Producer and Distributor of the IMP 25
7.1.3. Preparation + Dosage + administration of the IMP 25

7.1.4. Permitted dose adjustments and interruption of treatment 27
7.1.5. Duration of treatment..... 27
7.1.6. Packaging and Labeling of the IMP 27
7.1.7. Storage conditions of the IMP..... 27
7.1.8. Known side effects of the medication 27
7.2. Concomitant / Rescue Medication 29
8. Study Specific Procedures 29
8.1. Randomisation 29
8.2. Study specific interventions..... 29
8.3. Overview of collected data 30
8.4. Schematic overview of the data collection & interventions 31
8.5. Restrictions for subjects during the study 32
9. Sampling 32
9.1. Types and number of samples 32
9.2. Timepoints of sampling..... 32
9.3. Sample Handling & Analysis..... 32
9.4. Sample Storage and/or shipping..... 33
9.5. Future use of stored samples 33
10. Statistical Considerations 34
10.1. Sample size calculation 34
10.2. Type of statistical methods..... 34
10.3. Statistical analysis team 35
11. Data handling 35
11.1. Method of data collection..... 35
11.1.1. Case Report Form 35
11.1.2. Data directly collected in the CRF (no source available)..... 35
11.2. Data storage..... 35
11.3. Archiving of data 36
11.4. Access to data..... 36
Study Data, Data Ownership and Data Sharing with KCE 36
12. Safety..... 38
12.1. Definitions 38
12.2. Reporting requirements 39
12.2.1. AE reporting..... 39
12.2.2. SAE reporting 39

12.2.3. SUSAR reporting 39
12.3. List of contact details for safety reporting 40
12.4. Flowchart Reporting 40
12.5. Events, excluded from reporting 41
12.6. Data Safety Monitoring Board (DSMB) 41
12.7. Development Safety Update Report 41
13. Monitoring/Auditing/Inspection 42
13.1. Monitoring 42
13.1.1. General 42
13.1.2. Monitoring team 42
13.1.3. Scope 42
13.2. Inspection 42
13.3. Protocol Deviation policy 42
13.4. Serious breach to GCP and/or the protocol 42
14. Ethical and legal aspects 43
14.1. Good Clinical Practice 43
14.2. Informed Consent 43
14.3. Approval of the study protocol 43
14.3.1. General 43
14.3.2. Protocol amendments 44
14.4. Confidentiality and Data Protection 44
14.5. Liability and Insurance 44
14.6. End of Study Notification 44
15. Publication policy 45
16. Reference List 45

LIST OF ABBREVIATIONS

AE	=	Adverse Event
AECC	=	American-European Consensus Conference
ARDS	=	Acute Respiratory Distress Syndrome
CI	=	Coordinating Investigator
COVID-19	=	Coronavirus induced disease-2019
CT	=	Clinical Trial Unit
DSMB	=	Data Safety Monitoring Board
DSUR	=	Development Safety Update Report
EC	=	Ethics Committee
ECG	=	Electrocardiogram
ECMO	=	Extracorporeal Life Support Organisation
eCRF	=	electronic Case Report Form
EDC	=	Electronic Data Capture
EPD	=	Electronic Patient Dossier
FAMHP	=	Federal Agency for Medicines and Health Products
FiO2	=	Fraction of inspired oxygen
FPI	=	First Patient In
FVC	=	Forced vital capacity
GCP	=	Good Clinical Practice
GDPR	=	General Data Protection Regulation
GMP	=	Good Manufacturing Practice
HIRUZ	=	Health, Innovation and Research Institute UZ Ghent
HLH	=	Hyperferritinemia and Hemophagocytic Lymphohistiocytosis
IB	=	Investigator's Brochure
ICF	=	Informed Consent Form
ICH	=	International Council for Harmonisation
IL-1	=	Interleukin-1
IL-6	=	Interleukin-6
IMP	=	Investigational Medicinal Product
IMPD	=	Investigational Medicinal Product Dossier
LVLS	=	Last Visit, Last Subject
PCWP	=	Pulmonary Capillary Wedge Pressure
PEEP	=	Positive End Expiratory Pressure
PI	=	Principal Investigator
PaO_2	=	Partial pressure of oxygen
SAE	=	Serious Adverse Event
SAR	=	Serious Adverse Reaction
sHLH	=	secondary hemophagocytic lymphohistiocytosis
SmPC	=	Summary of Product Characteristics
SOP	=	Standard Operating Procedure
SUSAR	=	Suspected Unexpected Serious Adverse Reaction
TLC	=	Total Lung Capacity

1. Protocol Summary

COV-AID trial: Comparisons and combinations of IL-6 and IL-1 blockade in patients with acute hypoxic respiratory failure and systemic cytokine release syndrome due to COVID-19

Title	A prospective, randomized, factorial design, interventional study to compare the safety and efficacy of combinations of blockade of interleukin-6 pathway, and interleukin-1 pathway to best standard of care in improving oxygenation and short- and long-term outcome of COVID-19 patients with acute hypoxia and systemic cytokine release syndrome
Acronym	COV-AID: Treatment of **COV**ID-19 patients with **a**nti-**i**nterleukin **d**rugs
Protocol number	COV-AID
Protocol version	V3.0
EudraCT number	2020-001500-41
Clinicaltrials.gov number	When available
Sponsor	University Hospital Ghent
Co-ordinating Investigator	Bart N. Lambrecht
Type of study	Interventional
Phase	III
Purpose of study	To study the safety and effectiveness of individually or simultaneously blocking IL-6 and IL-1 versus standard of care on blood oxygenation and systemic cytokine release syndrome in patients with COVID-19 coronavirus infection and acute hypoxic respiratory failure and systemic cytokine release syndrome
Study design	2 by 2 factorial design<br>Prospective, multi-centre randomized, open label study
Primary objective	**Study if blockade of IL-6 +/- IL-1 to block the cytokine storm and acute lung injury in comparison with usual care reduces time to clinical improvement as defined by an increase of more than 2 on the 6 point ordinal scale or discharge from the hospital**
Primary endpoint	Time to clinical improvement (defined as the time from randomization to either an improvement of two points on a six-category ordinal scale (see below) measured daily or discharge from the hospital)<br>Time scale: score measured daily up to hospital discharge, death, or the end of the study, whichever comes first<br>1. Death<br>2. Hospitalized, on invasive mechanical ventilation or ECMO;<br>3. Hospitalized, on non-invasive ventilation or high flow oxygen devices;<br>4. Hospitalized, requiring supplemental oxygen<br>5. Hospitalized, not requiring supplemental oxygen<br>6. Not hospitalized

Number of participants	342
Study population and main inclusion criteria	Hospitalised adult patients with COVID-19 infection and acute hypoxia<br>Presence of hypoxia defined as ratio PaO_2/FiO_2 below 350 and signs of systemic cytokine release syndrome characterized by high serum ferritin, or high LDH or deep lymphopenia<br>Patients who have not been on mechanical ventilation for more than 24h before randomisation.<br>First treatment administration should happen as quickly as possible after randomisation.
Control arm	Standard of Care (SoC) (group A)
Experimental arms	SoC + Anakinra (Group B)<br>SoC + Siltuximab (Group C)<br>SoC + Siltuximab + Anakinra (Group D)<br>SoC + Tocilizumab (Group E)<br>SoC + Tocilizumab + Anakinra (Group F)
Investigational drug, dose, route	The study investigates IL-6 and IL-1 blockade. Current investigational drugs are listed below. If a new IL-6 or IL-1 blocker or other intervention becomes available, the data will be reviewed by the trial steering committee (TSC) and the protocol may be amended to include it.<br>Anakinra (KINERET®) once daily 100 mg SC, day1-28<br>Tocilizumab (ROACTEMRA®), single IV infusion, 8 mg/kg body weight<br>Siltuximab (SYLVANT®), single IV infusion, 11mg/kg body weight
Treatment duration	28 days<br>Anakinra: 28 days SC treatment<br>Tocilizumab: single IV injection<br>Siltuximab: single IV injection
Follow-up duration	10-20 weeks
Study duration	From first patient in to final report<br>12 months

2. Rationale and background

2.1. Rationale

Coronavirus disease 2019 (COVID-19), a respiratory tract infection inflicted by a new coronavirus SARS-CoV-2 was for the first time encountered in Wuhan, China in December 2019. It has now evolved to a pandemic threat with unknown outcome. Genetic sequencing of the virus suggests that it is a betacoronavirus closely linked to the SARS virus (1).

Most people with COVID-19 develop mild respiratory illness with upper airway symptoms, taste and smelling loss, cough, malaise and transient fever (Stage I of disease). In this stage of the disease, viral replication is high, and the immune system at this stage fights the infection. In a subgroup of patients, there is a second phase of the disease (stage II), that occurs after approximately 7-10 days and is accompanied by increasing respiratory symptoms, persistent fever, and shortness of breath. In this stage of the disease there can be no signs (stage IIa) or signs of hypoxia (stage IIb) on blood gas analysis. Occurrence of respiratory symptoms and dyspnea are a sign of acute lung injury, and patients will often have bilateral ground glass opacities on chest CT. The progression from stage I to later stages occurs in approximately 15% of patients, and requires hospitalization. A further 5% of patients develop stage III disease that requires admission to an intensive care unit mostly due to acute respiratory distress syndrome (ARDS), sepsis and septic shock, multiorgan failure, including acute kidney injury and cardiac injury (2). As patients progress from stage I to stage III, there are increasing signs of a systemic hyperinflammatory response, as reflected by increased levels of cytokines, CRP and ferritin, and some patients even develop frank secondary haemophagocytic lymphohistiocytosis (sHLH) characterized in decreasing lymphocytes, neutrophils and platelets, accompanied by diffuse intravascular coagulation.

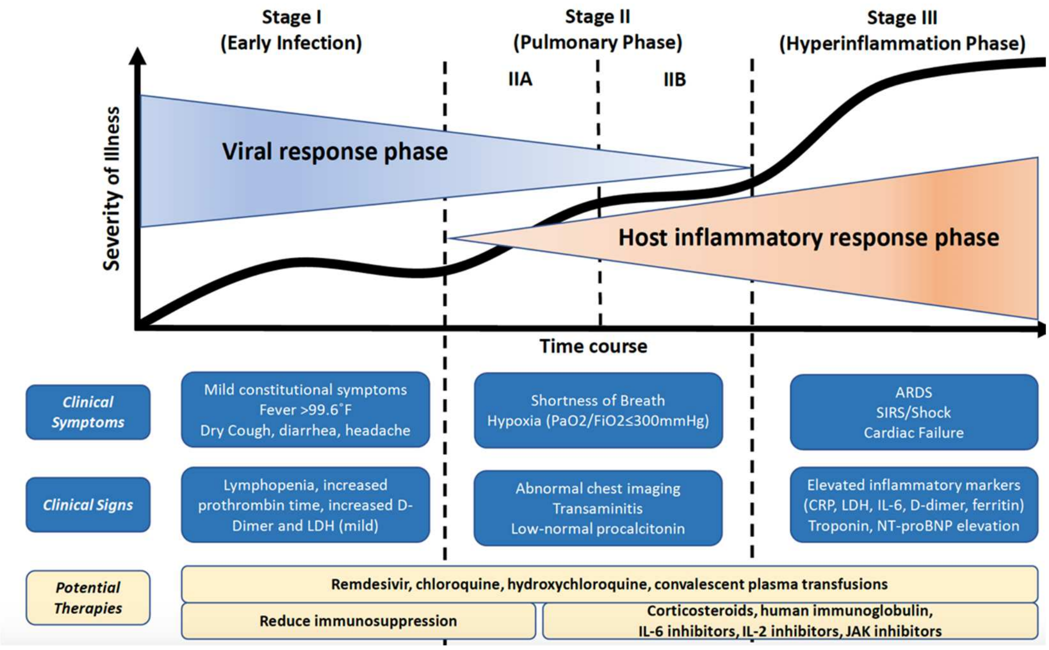

sHLH is an under-recognised, hyperinflammatory syndrome characterised by a fulminant and fatal hypercytokinaemia with multiorgan failure. In adults it is known that sHLH is most commonly triggered by viral infections and occurs in 3·7–4·3% of sepsis cases. Cardinal features of sHLH include unremitting fever, cytopenias, and hyperferritinaemia; pulmonary involvement can present as ARDS.

Viral, bacterial, and fungal pulmonary infections can all cause the cytokine storm syndrome that may be challenging to differentiate on clinical grounds. A complex cytokine response that builds in infection

is characterized by series of overlapping networks. Cytokines TNF and IL-1α and the chemotactic cytokines IL-8 and MCP-1 are indicative of an acute response that appear almost immediately after infection, followed by a more sustained increase in IL-6. Interactions between IL-6 and its soluble receptor enhance the activity of IL-6 on target cells to further aggravate inflammation. IL-6 production is stimulated by TNF and IL-1b, therefore a measurement of IL-6 concentration in peripheral blood has often been used to assess the intensity of systemic cytokine responses in patients with sepsis, providing an integrated readout signal of these two early-response cytokine. Compensatory repair processes are initiated soon after inflammation begins, in an attempt to restore tissue and organ function. IL-10, which is an anti-inflammatory cytokine is secreted as the body attempts to control the acute systemic inflammatory response. Systemic production of IL-10 following the onset of a cytokine storm can serve as a marker of counter-anti-inflammatory response that has been termed "immunoparalysis", in that it is associated with downregulation of neutrophil and monocyte function in the systemic circulation, and leads to downregulation of HLA-DR on monocytes. The same cytokines that cause the systemic response and are leading to rise in ferritin and CRP as biomarkers, are also profoundly involved in causing acute lung injury. Acute lung injury is accompanied by epithelial cell damage (loss of type 1 pneumocytes that line the alveolar space), initiation of the coagulation cascade (with endothelial and interstitial fibrin deposition) and activation of the complement cascade, which leads to further cell recruitment and perpetuation of damage.

A balance of pro- and anti-inflammatory mechanisms is critical for maintaining the immune homeostasis systemically and in the lung and if one or more of these regulatory mechanisms are absent or aberrantly regulated, then the outcome may contribute toward a cytokine storm and progression of acute lung injury to franc clinical ARDS.

Downregulation of systemic inflammation might be conceptually beneficial in controlling systemic responses to local infections. However, it has been suggested that patients who survive the initial cytokine storm but subsequently die, may be those who do not recover from immunoparalysis. Patients with persistent downregulation of HLA-DR (a marker of immunosuppression) on monocytes 3 to 4 days after the onset of severe sepsis and cytokine storm have a high mortality rate, suggesting a rationale for therapy to reverse immunosuppression under such circumstances.

2.2. Background

The proposed development plan was guided by three specific considerations:

1. **Supportive Scientific Rationale:**

Predictors of fatality from a recent retrospective, multicentre study of 150 confirmed COVID-19 cases in Wuhan, China, included elevated ferritin (mean 1297·6 ng/ml in non-survivors vs 614·0 ng/ml in survivors; p<0·001) and IL-6 (p<0·0001), suggesting that mortality might be due to virally driven hyperinflammation (*1, 2*). Respiratory failure from (ARDS) is the leading cause of mortality in COVID-19 (4,5). A cytokine profile resembling sHLH is associated with COVID-19 disease severity, characterised by increased interleukin (IL)-2, IL-7, granulocyte-colony stimulating factor, interferon-γ inducible protein 10, monocyte chemoattractant protein 1, macrophage inflammatory protein 1-α, and tumour necrosis factor-α (*2*).

Liu concluded by analyzing 69 severe type COVID -19 patients that on admission, the baseline levels of IL-6, CRP, LDH and ferritin were closely related to the severity of COVID-19, and the elevated IL-6 was significantly related to the clinical manifestation of severe type patients. The decrease of IL-6 was linked to treatment effectiveness, while the increase of IL-6 indicated disease exacerbation. There was mild variation in IL-2, IL-4, IL-10, TNF-a, IFN-γ before and after treatment, all of which fluctuated within the normal range.

In another cross-sectional study, 100 patients were included and divided into mild, severe or critical groups. Correlation of peripheral blood inflammation-related indicators with disease was criticality analyzed. Cut-off values for critically ill patients were speculated using ROC curve analyses. With following parameters such as age (R=-0.564, P67.5 years, IL2R >793.5U/mL, CRP >30.7ng/mL, ferroprotein >2252μg/L, WBC>9.5*10^9/L or NC >7.305*10^9/L, the progress of COVID-19 to critical stage should be closely observed and possibly prevented. They eventually state that as inflammation is closely related to severity of COVID-19, IL-6, TNFα and IL-8 might be promising therapeutic targets.
Similar analysis on cytokines and cells has been done on another group of 102 mild and 21 severe COVID-19 patients. Significant differences were noticed between the two groups in CD4 + T, CD8 + T, IL-6 and IL-10 with low levels of CD4+T and CD8+T and higher IL-6 and IL-10 levels in severe patients (12)

Interleukin 1 is known as an inducer of fever and induces CRP, which is often enhanced in patients with severe COVID-19. IL-1 is an important mediator of fever in autoinflammatory syndromes/inflammasomopathies such as NOMID, CAPS, and Still's disease. Interleukin-1 is also an important mediator in acute lung injury. Although less evidence supports high levels of IL-1 in COVID-19 patients, this could be due to the fact that IL-1 is hard to measure in the serum of these patients, and could better be addressed by measuring levels of IL-1RA. IL-1 blockade using anakinra is currently also suggested as an arm in the multicentre ReMapCAP trial in ICU patients, built in as a new arm addition to the existing trial, because of the emerging COVID-19 crisis. Re-analysis of data from a phase 3 randomized controlled trial of IL-1 blockade (anakinra) in sepsis with ARDS showed significant survival benefit in patients with hyperinflammation, without increased adverse events (*3*).

Preliminary data reported on 21 patients in an *open label* study report a favourable clinical evolution of tocilizumab treated patients, with reduction in fever, hyperferritinemia, cytopenia.
A multicentre, randomised controlled trial of tocilizumab (IL-6 receptor blockade, licensed for cytokine release syndrome), has been approved in patients with COVID-19 pneumonia and elevated IL-6 in China (ChiCTR2000029765). This 63 patient study has been completed, but formal analysis has not been published. A large 400 patient Adaptive Phase 2/3, Randomized, Double-Blind, Placebo-

Controlled Study Assessing Efficacy and Safety of Sarilumab (anti-IL6R) for Hospitalized Patients With COVID-19 versus best standard of care has been launched in stage2b patients with COVID-19 infection in the US by Sanofi/Regeneron. (ClinicalTrials.gov Identifier: NCT04315298). Primary endpoints in that study will be clinical evolution on a six score scale, and time to resolution of fever >48h. Important secondary endpoints include parameters of lung oxygenation (PaO2/FiO2), and clinical outcome (hospital stay, all-cause mortality at 28 days). The FDA has also approved the initiation of a double-blind, randomized phase III clinical trial (COVACTA) of tocilizumab for use in combination with standard of care. The trial is about to start.

Preliminary data obtained at Hospital St.-Pierre Brussels show that IL-6 levels are increased in a subset of COVID-19 Stage-3 patients, and that tocilizumab (TCZ) reduces key inflammatory parameters like CRP, while improving oxygenation.

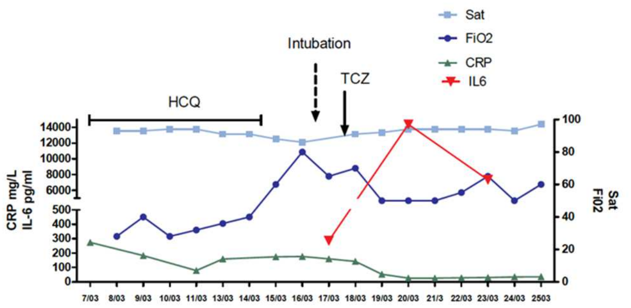

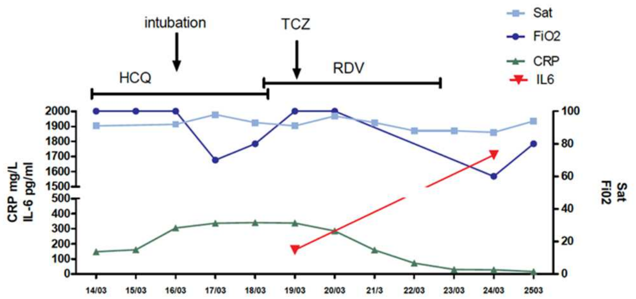

2. Experience: Use of IL-6R blockade is approved for treatment of cytokine release syndrome associated with CAR-T cell therapy, and has been widely used in long term treatment of RA and Still's disease. Anakinra has been used for treatment of systemic auto-inflammatory syndromes. Re-analysis of data from a phase 3 randomized controlled trial of IL-1 blockade (anakinra) in sepsis showed significant survival benefit in patients with hyperinflammation, without increased adverse events(*3*). Siltuximab has been used as an orphan drug indication for treatment of multicentric form of Castelman's disease (MCD), another auto-inflammatory syndrome(*4*)

3. Expediency: Toxicology, pharmacologic and safety data supports the immediate clinical use of IL-6 and/or IL-1 blockade in severe hypoxia and ARDS and in features of sHLH due to COVID-19 (*5, 6*). Investigator brochures of these drugs are available and contain detailed information on toxicity.

2.3. Risk/Benefit Assessment

COVID-19 poses a very significant risk of mortality of 3-7% and this percentage rises to mortality of 20% in patients with co-morbidity(*2, 7*). Of all infected patients, some 15-20% develop Acute lung

injury and severe respiratory symptoms necessitating hospital admission. Around 5% of infected patients will require invasive mechanical ventilation, and many of those (40-50%) will die. The current world-wide pandemic of COVID-19 is putting unforeseen stress on the entire primary, secondary and tertiary medical system, leading to unseen triage of patients that potentially benefit or not from admission to ICU units when they develop respiratory failure .

There are currently no treatments directed at improving gas exchange, cytokine release syndrome, and sHLH in COVID-19 patients, and no treatment that attempts to halt the progression from manageable acute hypoxic respiratory failure to ARDS (*5, 8-11*). Preventing such progression to ARDS could have a huge impact on the foreseeable overflow of the ICU units. We therefore believe the benefits of administering anti-IL6R and/or IL-1 blockade treatment in early stage COVID-19 acute hypoxic respiratory failure and signs of cytokine release syndrome outweighs the risks associated with a phase 4 IMP administration.

Anakinra was first approved for use in RA in the US in 2001 and subsequently in the EU in 2002. More than 3000 patients were involved in this development program. The initial IND for Anakinra was granted in 1991. The estimated cumulative exposure to Anakinra in completed company sponsored clinical studies up to May 1st 2018 is 6404 subject years in 8518 subjects with various indications. Since approval in 2001 the total post marketing exposure of Anakinra is > 102.000 patient years. Anakinra is administered s.c. at doses of 100 mg/day (RA) or in weight based dosing up to 8mg/kg/day in NOMID syndrome. In sepsis, several trials have used doses up to 2 mg/kg/hour IV over 72 hours to more than 500 patients, and were well tolerated. Re-analysis of data from a phase 3 randomized controlled trial of IL-1 blockade (anakinra) in sepsis showed significant survival benefit in patients with hyperinflammation, without increased adverse events(*3*).

Limitations and context

There is a large number of COVID-19 infected patients that are currently being hospitalized across the globe. By 30 march 2020, over 4524 patients have been admitted to Belgian hospitals with severe respiratory symptoms, and 513 people have died and 927 are admitted to the ICU, most likely all with severe acute lung injury. Worldwide, more than 31680 people have died as of March 29. We therefore believe that given the current ascending part of the epidemiology curve, with numbers of patients rising sharply, there will be no shortage of patients that are eligible.

There is currently a lot of pressure on using the IMPs as off-label indications to treat COVID-19 worldwide. We have checked availability of these drugs for investigational use with Belgian representatives of the company and have confirmation from ROCHE, EUSAPHARMA and SOBI that sufficient drug will be reserved for purchase as trial medication for this trial. A recent WHO ad hoc informal consultation on the use of IL-6/IL-1 antagonists in the clinical management of COVID19 infection convened on march 25th 2020 in Geneva and concluded that if we are to understand the real value of these immunomodulatory therapies and understand their risks and benefits, the limited stock of drugs should best be used to perform randomized controlled trials.

2.4. Primary Objectives

Justification for our objective is that preventing cytokine release syndrome and progression from early hypoxic respiratory failure and mild acute lung injury to ARDS could have a huge impact on the foreseeable overflow of the ICU units. The outcome of our study could thus have large impact from a medical, ethical and economic perspective.

The **hypothesis of the proposed intervention** is that IL-6 and/or IL-1 are important mediators of the cytokine release syndrome that has an important impact on acute lung injury and development of secondary cytopenias post COVID-19, and thus affect clinical outcome of the patients

the **primary objective** of this intervention is :
Study if blockade of IL-6 +/- IL-1 to block the cytokine storm and acute lung injury in comparison with usual care reduces time to clinical improvement as defined by an increase of more than 2 on the 6 point ordinal scale or discharge from the hospital

2.5. Secondary Objectives

-to investigate whether treatment with either tocilizumab, siltuximab, anakinra or combinations thereof

- -improves oxygenation
- -causes defervescence, measured as time to first fever-free 48h period
- -improves features of secondary haemophagocytic lymphohistiocytosis
- -improves features of secondary haemophagocytic lymphohistiocytosis in relation to serum IL-6 and IL-1
- -affects clinical outcome in relation to IL-6 and IL-1 levels
- -affects the rate of nosocomial infection
- -affects progression to mechanical ventilation, high oxygen delivery device, and/or ARDS in non-ventilated patients
- -affects length of dependency of ventilation in ventilated patients
- -affects all-cause mortality rate at 4 and 20 weeks post inclusion
- -affects long term 10-20 week follow up clinical status and lung function
- -is safe (number of AEs/SAEs/SARs/SUSARs)
- -When there is a significant association between IL-6 blockade and time to clinical improvement, tocilizumab and siltuximab will be compared versus usual care separately with respect to the primary endpoint.

3. End Points + Time Points

3.1. Primary End Points + Time Points

Time to clinical improvement (defined as the time from randomization to either an improvement of two points on a six-category ordinal scale measured daily till day 28 or discharge from the hospital or death)

1. Death
2. Hospitalized, on invasive mechanical ventilation or ECMO;
3. Hospitalized, on non-invasive ventilation or high flow oxygen devices;
4. Hospitalized, requiring supplemental oxygen
5. Hospitalized, not requiring supplemental oxygen
6. Not hospitalized

3.2. Secondary End Points + Time Points

-Time since randomization until improvement in oxygenation, defined as independence from supplemental oxygen

-Mean change in oxygenation defined by Pa02/FiO2 while breathing room air between day 1 and day 15 (or hospital discharge, whichever is first)

-Number of days with hypoxia

-Number of days of supplemental oxygen use

-Time since randomization until absence of fever for more than 48h without antipyretics

-Number of days with fever

-Time since randomization until halving of CRP levels compared to peak value during trial

-Time since randomization until halving of ferritin levels compared to peak value during trial

-Incidence of AEs/SAEs/SARs/SUSARs during 28 days

-Duration of hospital stay

-Duration of hospital stay in survivors

-Mean change in clinical sign score between day 1 and day 7 and between day 1 and day 15 (or on discharge, whichever is first)

-Time since randomization until clinical sign score <6 maintained for 24h

-Mean change of SOFA score between day 1 and day 7 or between day 1 and day 15 (or on discharge, whichever is first)

-Mean change NEWS2 score between day 1 and day 7 or between day 1 and day 15 (or on discharge, whichever is first)

-Time since randomization until NEWS2 score less than 2 for at least 24h

- Percentage of patients reporting each severity rating on a 6-point ordinal scale
6-point Ordinal Scale at 15 days, in relation to serum IL-1 and IL-6

- Incidence of nosocomial bacterial or invasive fungal infection for 28 days after enrolment in trial

- incidence of secondary haemophagocytic lymphohistiocytosis defined by Hs score
Cardinal features of sHLH include unremitting fever, cytopenias, hyperferritinaemia, hypertriglyceridemia, pulmonary involvement can present as ARDS. Hs score calculation see http://saintantoine.aphp.fr/score/

-**Incidence of secondary haemophagocytic lymphohistiocytosis defined by Hs score** (Hs score calculation see http://saintantoine.aphp.fr/score/) **in relation to serum IL-1 and IL-6**

-Time since randomization until first use of high-flow oxygen devices, non-invasive or invasive mechanical ventilation in non-ventilated patients

-Time since randomization until first use of salvage systemic steroids in ventilated patients

-Ventilator-free days over 28 days from inclusion date

-Duration of mechanical ventilation in ventilated patients

-Duration of ICU stay in patients that enrolled in trial while already on invasive or non-invasive mechanical ventilation

-Time to progression to ARDS in ventilated patients

criteria-defined ARDS (according to the American-European Consensus Conference (AECC) diagnostic criteria for ARDS: acute onset; ratio of partial pressure of arterial oxygen to fraction of inspired oxygen (PaO_2/FiO_2) of 200mmHg or less, regardless of positive end-expiratory pressure; bilateral infiltrates seen on frontal chest radiograph; and pulmonary artery wedge pressure of 18 mm Hg or less when measured, or no clinical evidence of left atrial hypertension)

-**Time to progression to ARDS in ventilated patients according to IL-1 and IL-6**

-**All-cause mortality rate at 28 days post inclusion (excluding group that entered during ventilation)**
-**Percentage of patients in clinical status on 6-point Ordinal Scale at 10-20 weeks follow up**
-Incidence of lung function abnormalities at 10-20 weeks follow up
-**Incidence of lung fibrosis on chest CT scan at 10-20 weeks follow up**
-All cause mortality at 20 weeks post inclusion for the entire study population

4. Study design

4.1. Description of study design

This is a multi-center, interventional, open label, 6-arm 2x2 factorial design study designed to investigate the efficacy of **tocilizumab, tocilizumab and anakinra, siltuximab, siltuximab and anakinra and anakinra alone versus usual care** in improving short- and long-term outcome of COVID-19 patients with hypoxia and signs of cytokine release syndrome. A 2x2 factorial design was selected because it allows to answer two research questions simultaneously, while minimizing the number of patients enrolled in the trial, which is important in view of the emerging shortage of interleukin-blocking therapies. In addition, the number of patients not receiving study medication is kept to a minimum, which is more justifiable in such a severe disorder as COVID-19.

There are currently no treatments directed at halting the cytokine storm and acute lung injury to stop the progression from manageable hypoxia to frank respiratory failure and ARDS(*5, 9*). Preventing progression from early acute hypoxia and cytokine release syndrome to frank hypoxic respiratory failure and ARDS could have a huge impact on the foreseeable overflow of the ICU units, that is already happening in some countries and is bound to happen on a global scale. In ventilated patients, preventing the onset of ARDS, or shortening ICU stay could also be crucial in this regard. The study will be performed in adults, because several of the patients will have to be admitted to the intensive care unit. In pediatric patients, the presence of cytokine has been less described, and paediatric ICU units currently have limited experience and numbers of COVID-19 patients with cytokine release syndrome.

		**IL-6 blockade** (1:2 randomization)		
		No (n=114)	Yes (n=228)	
**IL-1 blockade** (2:1 randomization)	No (n=228)	Usual care (n=76)	Siltuximab (n=76)	Tocilizumab (n=76)
	Yes (n=114)	Anakinra (n=38)	Anakinra + siltuximab (n=38)	Anakinra + tocilizumab (n=38)

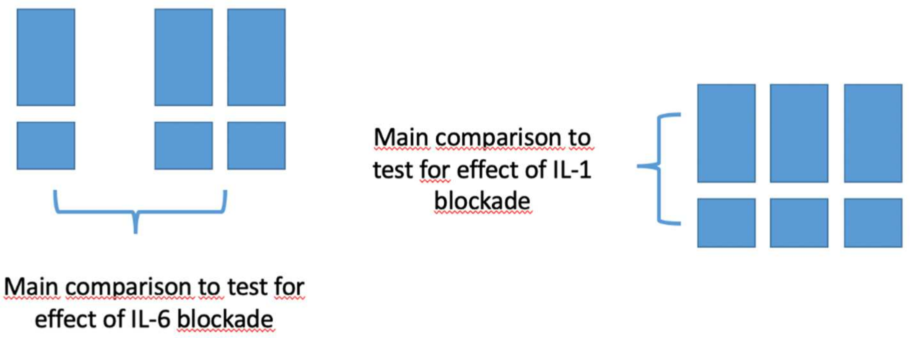

		RANDOMIZATION 2		
		Usual Care	Sylvant (Situximab)	Roactemra (Tocilizumab)
RANDOMIZATION 1	Usual Care	A: Usual Care (2/9)	C: Sylvant (2/9)	E: Roactemra (2/9)
	Kineret (Anakinra)	B: Kineret (1/9)	D: Kineret + Sylvant (1/9)	F: Kineret + Roactemra (1/9)

To measure the effectiveness of **tocilizumab, tocilizumab and anakinra, siltuximab, siltuximab and anakinra and anakinra versus usual care** on restoring lung homeostasis, we will assess the time to clinical improvement as defined as the time from randomization to either an improvement of two points on a six-category ordinal scale or discharge from the hospital using single IV injection (siltuximab or tocilizumab) combined or not with daily subcutaneous injections of anakinra until 28 days or hospital discharge, whichever is first. During the treatment period, we will perform daily clinical assessments of severity, daily laboratory check-up, measurements of oxygen saturation (pulse oximetry) in relation to FiO2, regular arterial blood gas measurements, regular chest X-rays, chest CT scans on clinical indication.

4.2. End of Study Definition

4.2.1. For an individual subject

The subject has completed the study if he or she has completed all phases of the study, including the last visit (week 10-20 clinical follow up visit) or the last scheduled procedures, as described in this protocol (see section "8. Study Specific Procedures").
For the primary endpoint, the last patient will be followed until the event has been observed or until 28 days after randomisation, whichever comes first.

4.2.2. For the whole study

Overall, the end of the study is reached when the last study procedure for the last subject has occurred: last subject, last visit (LSLV).
As soon as the whole study has ended (cfr. the definition above), the co-ordinating Investigator shall notify the HIRUZ Clinical Trial Unit, so that the Competent Authority and the Ethics Committee can be informed in a timely manner according to the regulatory requirements (within 90 days after end of the study, or if the study had to be terminated early, this period must be reduced to 15 days and the reasons should clearly explained).

4.3. Estimated duration of the study

There is a large number of COVID-19 infected patients that are currently being hospitalized across the globe. In just 15 days time, the COVID-19 ward at Ghent University Hospital has admitted 85 confirmed cases, of which a significant portion (30%) already fulfilled eligibility criteria for the current proposed protocol. We estimate the study to terminate in 32 weeks, including last clinical follow up visits.

5. Inclusion and Exclusion Criteria

5.1. Inclusion Criteria

The following patients will be enrolled

-Recent (≥6 days of flu-like symptoms or malaise yet ≤16 days of flu-like symptoms or malaise prior to randomization) infection with COVID-19.Confident COVID-19 diagnosis confirmed by antigen detection test and/or PCR and/or positive serology, or any emerging and validated diagnostic laboratory test for COVID-19 within this period.
-In some patients, it may be impossible to get a confident laboratory confirmation of COVID-19 diagnosis after 24h of hospital admission because viral load is low and/or problems with diagnostic sensitivity. In those cases, in absence of an alternative diagnosis, and with highly suspect bilateral ground glass opacities on recent (<24h) chest-CT scan (confirmed by a radiologist and pulmonary physician as probable COVID-19), and a typical clinical and chemical diagnosis with signs of cytokine release syndrome, a patient can be enrolled as probable COVID-19 infected. In all cases, this needs confirmation by later seroconversion.
-Presence of hypoxia defined as

PaO2/FiO2 below 350 while breathing room air in upright position or PaO2/FiO2 below 280 on supplemental oxygen and immediately requiring high flow oxygen device or mechanical ventilation.

<u>Estimating FiO2 for nasal canula, face mask or face mask + reservoir</u>

Method	O_2 flow (l/min)	Estimated FiO2 (%)
Nasel cannula	1	24
	2	28
	3	32
	4	36
	5	40
	6	44
Nasopharyngeal catheter	4	40
	5	50
	6	60
Face mask	5	40
	6-7	50
	7-8	60
Face mask with reservoir	6	60
	7	70
	8	80
	9	90
	10	95

-signs of cytokine release syndrome defined as

ANY of the following

-serum ferritin concentration >1000 mcg/L and rising since last 24h
-single ferritin above 2000 mcg/L in patients requiring immediate high flow oxygen device or mechanical ventilation
-lymphopenia defined as <800 lymphocytes/microliter and two of the following extra criteria
Ferritin > 700 mcg/L and rising since last 24h
-increased LDH (above 300 IU/L) and rising since last 24h
-D-Dimers > 1000 ng/mL and rising since last 24h
-CRP above 70 mg/L and rising since last 24h and absence of bacterial infection
-if three of the above are present at admission, no need to document 24h rise

-Chest X-ray and/or CT scan showing bilateral infiltrates within last 2 days
-Admitted to specialized COVID-19 ward or an ICU ward taking care of COVID-19 patients
-Age ≥ 18 years
-Male or Female
- Women of childbearing potential must have a negative serum pregnancy test pre-dose on day 1. Women of childbearing potential must consistently and correctly use (during the entire treatment period and 3 months after last reatment) 1 highly effective method for contraception.
-Willing and able to provide informed consent or legal representative willing to provide informed consent

5.2. Exclusion Criteria

-Patients with known history of serious allergic reactions, including anaphylaxis, to any of the study medications, or any component of the product.
-**mechanical ventilation > 24 h at randomization**
-clinical frailty scale above 3
-active bacterial or fungal infection
-unlikely to survive beyond 48h
-neutrophil count below 1500 cells/microliter
-platelets below 50.000/microliter
-Patients enrolled in another investigational drug study
-patients on high dose systemic steroids (> 8 mg methylprednisolone or equivalent for more than 1 month) for COVID-19 unrelated disorder
-patients on immunosuppressant or immunomodulatory drugs
-patients on current anti-IL1 or anti-IL6 treatment
-signs of active tuberculosis
-serum transaminase levels >5 times upper limit of normal, unless there are clear signs of cytokine release syndrome defined by LDH >300 IU/L and ferritin >700 ng/ml
-history of (non-iatrogenic) bowel perforation or diverticulitis
- Pregnant or breastfeeding females (all female subjects deemed of childbearing potential by the investigator must have negative pregnancy test at screening)

5.2.1. Screen failures

Screen failures are defined as subjects who consent to participate in the clinical study but are not subsequently randomly assigned to the study intervention or entered in the study. A minimal set of screen failure information will be kept to ensure transparent reporting of screen failure subjects.

6. Target Population

6.1. Subjects

6.1.1. Number of subjects and planned recruitment rate

There is a large number of COVID-19 infected patients that are currently being hospitalized across the globe. In just 14 days time, our COVID-19 ward at Ghent University Hospital has admitted 91 confirmed cases, of which a significant portion (30%) already fulfil eligibility criteria for the current proposed protocol. Similar numbers of patients are currently being seen in the participating centers in Belgium. We therefore believe that given the current ascending part of the epidemiology curve, with numbers of patients rising sharply, there will be no shortage of patients that are eligible.

342 patients will be recruited within an accrual period of 8 weeks in order to observe 215 patients with clinical improvement (when the last patient has 28 days of follow-up).
We expect to recruit 100 patients / week over all centers.

6.1.2. Withdrawal and replacement of subjects

Subjects are free to withdraw from participation in the study at any time upon request.
An investigator may discontinue further administration of study drug for the following reasons (however assessments will continue to be made and patients will remain in the intent to treat population for statistical analysis):

- Allergic reactions (anaphylactic shock) to study drugs
- Pregnancy
- Duration of mechanical ventilation has moved beyond >24h at time of randomization
- Significant study intervention non-compliance
- If any clinical adverse event (AE), laboratory abnormality, or other medical condition or situation occurs such that continued participation in the study would not be in the best interest of the subject
- If the subject meets an exclusion criterion (either newly developed or not previously recognized) that precludes further study participation

The investigator can exceptionally discontinue further study procedures if they feel that the patient is too sick and that it wouldn't be ethical to continue treatment. However, all safety data must be collected.

In all cases, the reason why subjects stopped study medication must be recorded in detail in the eCRF and in the subject's medical records.

The following actions must be taken if a subject fails to return to the clinic for a required study visit (visit at 10-20 weeks post end of study) :

- The site will attempt to contact the subject and reschedule the missed visit within 4 weeks and counsel the subject on the importance of maintaining the assigned visit schedule and ascertain if the subject wishes to and/or should continue in the study.
- Before a subject is deemed lost to follow-up, the investigator or designee will make every effort to regain contact with the subject (where possible, 3 telephone calls and, if necessary, a

certified letter to the subject's last known mailing address or local equivalent methods). These contact attempts should be documented in the subject's medical record or study file.

• Should the subject continue to be unreachable, he or she will be considered to have withdrawn from the study with a primary reason of lost to follow-up.

6.2. Method of recruitment

Subjects will be recruited at the COVID-19 hospitalization ward and or ICUs at the participating centers. The study will be proposed by the treating physician to all subjects with confirmed COVID-19 infection and a presence of hypoxia, and signs of cytokine release syndrome.

There will be no compensation for study participation. Study medication is paid for by Kennis Centrum KCE, and dispatched from UZ Gent Hospital Pharmacy.

Since this is a hospital based trial, in which patients are severely ill and in infection quarantine, we suspect the retention in the trial to be high.

6.3. Screening

Patients will be informed about the study by the treating physician.
After receiving full explanation, having received sufficient time to considerer the trial, asking questions and receiving satisfying responses to all questions, patients will be asked to sign ICF.

A serum pregnancy test will be done (female patients only).
Medical history will be checked for review of exclusion criteria and relevant subject information.

Patients will be continuously monitored on the COVID-19 ward.
Exams (standard of care) include, but are not limited to:

- ECG
- Chest X-Ray, and/or CT-scan
- Laboratory tests for leukocyte formula, kidney and liver function, ferritin levels, LDH level
- Vital signs
- Pulse oximetry, Arterial blood gas, capnography

As soon as all in- and exclusion criteria are checked and patient is considered eligible, patient can be randomized in IVRS. This is allowed the day before D1 in order to make practical arrangements to start treatment.

7. Investigational Medicinal Product (IMP)

7.1. Name of the IMP

7.1.1. Composition and active substance of the IMP

7.1.1.1 Tocilizumab (ROACTEMRA®) is a humanised anti-IL6 antibody approved for the treatment of rheumatoid arthritis, juvenile idiopathic arthritis and giant cell arteritis. It is also licensed for the induction of the rapid reversal of cytokine release syndrome (CRS), a form of cytokine storm caused by CAR-T treatment. Il-6 is a key pro-inflammatory cytokine and an important mediator of fever and the acute phase response. Tocilizumab prevents Il-6 from binding to soluble and cell associated Il-6 receptors inhibiting signalling. **7.1.1.2 Siltuximab** (SYLVANT®) is a chimeric antibody neutralizing IL-6. It has been used in treatment of metastatic kidney cell tumors, prostate cancer and multicentric form of Castelman's disease.

7.1.1.3 Anakinra (KINERET®) is an IL-1 inhibitor binding to the IL-1 receptor. It is indicated in EU for treatment of Rheumatoid arthritis, cryopyrin-associated periodic syndromes (CAPS, NOMID) and Still's disease, a rare disease causing inflammation of joints as well as rash and fever.

7.1.2. Producer and Distributor of the IMP

Anakinra (KINERET®) will be purchased and distributed from SOBI
Tocilizumab (ROACTEMRA®) will be purchased and distributed from ROCHE
Siltuximab (SYLVANT®) will be purchased and distributed from EUSAPHARMA

7.1.3. Preparation + Dosage + administration of the IMP

Drugs will be purchased by the central hospital pharmacy of UZ Gent and dispatched to other participating centers.

Preparation and storage:
Roactemra® (tocilizumab) IV- Infusion prepared starting from flasks containing 400mg/20ml stock solution (to dilute for infusion) Source SKP FAGG
Shelf life : Diluted product: After dilution the prepared infusion solution is chemically and physically stable in sodium chloride 9 mg/ml (0,9 %) injection solution at 30°C for a period of 24 hours. For safety reasons, the infusion solution should be used immediately to avoid growth of microbes. When injection cannot be done immediately, product quality and storage conditions are the responsibility of the person who prepared the solution, and the diluted product should not be stored longer than 24 hours at a temperature between 2-8°C. If product was diluted under controlled and validated aseptic conditions, storage time can be extended.
Shelf life Closed injection vial: 30 months
Store injection vials refrigerated (2°C – 8°C). Do not freeze-thaw.
Keep injection vials away from direct sun light by storing them in the original packing.

Due to shortage of ROACTEMRA® for IV usage, some hospital will dispense ROACTEMRA® for subcutaneous route in infusion bags for IV injection, as per instructions of the manufacturer:

Roactemra® (tocilizumab) IV infusion prepared from Prefilled syringe 162mg / 0.9ml SC
Source : Emergency use of Actemra SC in an infusion bag (see attachment) + SKP FAMHP
Shelf life : Diluted product: At a concentration of 1.6 - 8.8 mg / ml in a sodium chloride 0.9% 100ml infusion: can be used up to 9 hours after dilution (including administration time of max. 2 hours)
Unused Syringe should be stored at 2-8 ° C. Dose solutions using aseptic technique were prepared in 100 mL IV infusion bags containing normal saline (0.9% NaCl) and constructed with product contacting materials of polyvinyl chloride (PVC), polyolefin (PO), polyethylene (PE) and/or polypropylene (PP) by administering the necessary number of RoActemra 162 mg solution for injection in pre-filled syringes into an infusion bag to achieve the desired dosage to be administered. Example: 4 syringes of RoActemra/Actemra 162 mg solution for injection in pre-filled syringe administered into a 100mL i.v. infusion bag would yield a dose equivalent to 8mg/kg for a patient weighing 81 kg.

When taken out of refrigerated storage conditions, RoActemra should be administered within 8 hours and should not be stored above 30 ° C.

Sylvant® (Siltuximab) 400mg powder concentrate
Source SKP FAMHP (FAGG)
Shelf life : Diluted product: 8 hours shelf life at room temperature (after reconstitution)
Vial should be dissolved within 60min, further dilution should be done within 2 hours

Vial: Store refrigerated (2 °C - 8 °C).

Kineret® (Anakinra) 100mg pre-filled syringe
Source: SKP FAMHP (FAGG)
Shelf life : Store refrigerated (2 ° C - 8 ° C).
For ambulatory use, Kineret® may be removed from the refrigerator for a period of 12 hours at temperatures up to 25 ° C, without exceeding the expiration date. At the end of this period, the drug should not be put back in the refrigerator and should be destroyed.

Dosing and administration :

Dose justification
In this trial, we are targeting patients with COVID-19 and signs of a beginning cytokine storm (reflected by increasing ferritin, CRP, LDH and D-dimers and declining lymphocytes). In this phase of the disease, the levels of cytokines IL-1 and IL-6 are still in the range also found in patients with rheumatoid arthritis. Therefore, the dosing of the IMPs is taken from the SKP FAMHP, by analogy with indications in rheumatoid arthritis and Castelman's disease.

ROACTEMRA®

will be given via single IV infusion at a dose of 8 mg/kg with a maximum infusion of 800 mg/injection 8mg / kg (= 0.4ml / kg) in an infusion of 100ml NaCl 0.9% and administration over 1 hour. If Roactemra® is directly prepared from the vials for IV injection: no further specifications regarding the use of a 0.2 or 0.22 filter when administered. When the Roactemra® IV infusion bag is prepared from prefilled syringes , use a PO, PE, PP, PBD or PUR infusion line with 0.2 or 0.22 µm PES or PS filter during administation. As a measure of precaution, an inline filter is mandatory.

SYLVANT ®
Will be given via single IV infusion at a dose of 11 mg / kg given over 1 hour as an intravenous infusion in glucose 5% (Ad-preparation: the same volume of product must be withdrawn from the infusion before adding the Sylvant®)
Administer via using PVC, or polyurethane (PU), or PE coated administration sets with a 0.2-micron in-line polyethersulfone (PES) filter.

KINERET®
Will be given 1x / day 100 mg SC 28 days (or until discharge from hospital)
Do not shake. Allow the pre-filled syringe to reach room temperature before the injection.
Subcutaneous injection; It is recommended that the injection site would be varied to avoid discomfort at the site of injection. Cooling the injection site, prewarming/preheating the injection liquid to reach room temperature, use of cold packs (before and after the injection) and use of topical glucocorticoids and antihistamines after injection may reduce the signs and symptoms of the reaction at the injection site.

7.1.4. Permitted dose adjustments and interruption of treatment

No dose adjustments and interruptions are permitted during this trial. In case of anaphylaxis or severe AE, the drug will be immediately interrupted. Since anaphylaxis to protein drugs like tocilizumab or siltuximab or anakinra is hard to predict and can occur already to very low doses in case of prior anaphylaxis, we will not attempt a subsequent dose reduction.

7.1.5. Duration of treatment

Maximum 28 days
Anakinra: max 28 days SC treatment (or until hospital discharge whichever comes first)
Tocilizumab: single IV injection
Siltuximab: single IV injection

7.1.6. Packaging and Labeling of the IMP

The IMPs will be purchased from commercial stocks of manufacturers and dispatched to all participating centers by the central hospital pharmacy of UZ Gent. Study medication will be relabelled at the pharmacy of every participating centers per instruction and approval of FAGG.

7.1.7. Storage conditions of the IMP

All study medication should be stored between 2 and 8 degrees Celsius. Refer to SMPC for stability after reconstitution.

7.1.8. Known side effects of the medication

Tocilizumab (ROACTEMRA®): Increased serum cholesterol, increased serum alanine aminotransferase, increased serum aspartate aminotransferase, infusion-related reaction.. For full list of side effects see package leaflet insert.

Siltuximab (SYLVANT®): Edema (cardiovascular), pruritus, skin rash, weight gain, hyperuricemia, localized edema, upper respiratory tract infection. For full list of side effects see package leaflet insert.

Anakinra (KINERET®): The most common side effects (which may affect more than 1 patient in 10) are headache, injection site reactions (redness, bruising, pain and inflammation), and increase in blood cholesterol. For the full list of side effects of KINERET®, see the package leaflet. KINERET® must not be used in people who are hypersensitive (allergic) to anakinra, to any of the other ingredients, or to proteins produced by Escherichia coli (a type of bacterium). KINERET® must not be started in patients who have neutropenia.

7.2. Concomitant / Rescue Medication

There are no restrictions regarding concomitant/rescue medication.

8. Study Specific Procedures

Patients will be informed about the study by the treating physician.
After receiving full explanation, having received sufficient time to considerer the trial, asking questions and receiving satisfying responses to all questions, patients will be asked to sign ICF.
The ICF process will be performed before any other study related procedure.

8.1. Randomisation

In this open label trial patients will be randomized using REDCap (electronic IVRS system).
We will use stratified permuted block randomization with varying block sizes. We will stratify according to center. The randomization will be done separately for the two main comparisons to reflect the factorial nature of the trial.
For the comparison of Anakinra versus usual care, the allocation ratio is 1:2 (more patients in the usual care group). For the comparison of IL6 blockers (SYLVANT® and ROACTEMRA®) versus usual care, the allocation ratio is 1:2 (more patients on IL6 blockers). Within the group on IL6 blockers, there is equal allocation to SYLVANT® and ROACTEMRA® (1:1).
Having two separate randomization schemes implies we cannot guarantee the perfect randomization ratios. However, it will allow for more flexibility in case not all centers have access to all drugs and it will reduce the maximum imbalance between two groups.

8.2. Study specific interventions

This is a hospital based intervention trial, in which patients with COVID-19 will be randomized to be treated with **tocilizumab, tocilizumab and anakinra, siltuximab, siltuximab and anakinra, anakinra or usual care.** Patients with COVID-19 infection and respiratory failure are severely ill, and will require multiple daily clinical exams, blood sampling (including blood procalcitonin levels), vital parameter measurements, blood oxygenation measurements, and chest X-rays. These are all part of the clinical management plan of the patients, and data stored in the electronic patient file will be used as part of the assessment of efficacy and safety profile of the study drugs.

On screening, a blood sample will be taken, preferentially during routine blood sampling, to determine exclusion criteria (pregnancy, high ferritin level, LDH, D-dimers, blood lymphocyte counts, transaminases).

On day 1, prior to **tocilizumab, tocilizumab and anakinra, siltuximab, siltuximab and anakinra and anakinra** treatment, two tubes of blood serum (5 ml) and four tubes of EDTA tube (10 ml) will be collected for measuring blood cytokine and chemokine levels, and activation of immune cells. Also, an arterial blood gas determination via arterial puncture will be taken.

On day 6, on day 15 (or on discharge, whichever is first) two tubes of blood serum (5ml) and four tubes of EDTA tube (10 ml) will be collected for measuring blood cytokine and chemokine levels, and activation of immune cells. Also, an arterial blood gas determination via arterial puncture will be taken.

On days 1, patients in various groups will receive single IV injection of SYLVANT® 11mg/kg or ROACTEMRA® 8mg/kg (max dose 800 mg), on top of standard of care.

On days 1-28, (or until hospital discharge, whichever comes first), some patients in groups will additionally receive daily injection of 100 mg KINERET® subcutaneously. If kidney function falls below 30 ml/min GFR, dosing needs to be adjusted to 100mg once every other day (q2d).

On a final clinical visit between week 10-20 two additional serum tube (5ml) and four EDTA tubes (10 ml) will be taken.

8.3. Overview of collected data

1. patient demographics
   age, sex, day of admission, date of randomisation, date of discharge
2. day of COVID-19 positivity, and conversion to negative test if available
3. patient biometry
   weight, length, BMI
4. Clinical and laboratory parameters on screening day and during trial
   -first day of illness (upper airway symptoms, fever, dyspnea) , potential source of infection
   -clinical examination findings (cyanosis, crepitation's and rales, heart murmurs, peripheral edema)
   -vital signs (temperature, blood pressure, heart rate, breathing rate)
   -pulse oximetry data (SaO2)
   -clinical blood gas sampling (PaO2, PaCO2, HCO3) measured in prone position while breathing room air
   -clinical chemistry sampling (ferritin, procalcitonin levels (daily), leukocyte formula, platelets, kidney and liver function, D-dimers, triglycerides)
   -Chest X-ray and/or CT characteristics and radiology clinical report
   -in case of admission to ICU : invasive monitoring data (arterial blood pressure, PCWP, continuous O2 saturation, continuous ECG, ventilatory parameters (tidal volume, FiO2, PEEP pressure, peak pressure, minute ventilation)
   -mortality and date of death
5. All standard care drugs used during the trial and on day of enrolment of the trial, including oxygen flow rate.
6. Basic clinical data on prior medical history (prior lung diseases, smoking history, prior lung function measurements (preferentially within 5 preceding years), prior gas exchange measurements and medication use will be collected from electronic medical record.
7. Status of the patient on the 6 level ordinal scale needs to be assessed and recorded every day
8. Study specific measurements (see table). The eCRF will be checked as to ensure that all data needed to assess the secondary endpoints are collected.

8.4. Schematic overview of the data collection & interventions

**Procedures**	Screening	D1	D6 Or discharge (whichever comes first)	D15 Or discharge (whichever comes first)	Discharge Only if >15d	Follow-up (10-20w after start treatment)
Informed consent	X					
Inclusion/exclusion criteria check	X					
Randomisation°		X°				
Medical History	X					
Lung function[1]						X
Physical examination[1]	X	---------------------------------------→				X
ECG[1]	X	----------------------------→			X	
Arterial blood gas	X[1]	X[2]	X[2]	X[2]	X[2]	X[2]
Chest X-ray or CT scan[1]	X	----------------------------→			X	x
Laboratory assessments[1]	X	---------------------------------------→				X
Procalcitonin	X[5]	Daily until D28/discharge whichever comes first				
Blood sampling 2x 5ml Serum		X	X	X		X
Blood sampling 4x 10ml EDTA **(OPTIONAL, only in selected centres)**		X	X	X		X
Serum pregnancy test	X					
Vital signs[1]	X	---------------------------------------→				X
Breathing frequency[1]	X	---------------------------------------→				X
Pulse oximetry[1]	X	---------------------------------------→				X
six-category ordinal scale	X[4]	---------------------------------------→				
IMP[3]		(X) →				
Concomitant medications	X	---------------------------------------→				X
Adverse event check	X	---------------------------------------→				X

°As soon as all in- and exclusion criteria are checked and patient is considered eligible, patient can be randomized in IVRS. This is allowed the day before D1 in order to make practical arrangements to start treatment.

[1] per standard of care, information to be collected if available.

[2] Mandatory: patient in upright position, after minimal 3 minutes without supplemental oxygen. In case of inability to sit upright: same position is to be used for all measurements of PaO_2. In ventilated patients or ECMO patients PaO_2 can be taken from invasive arterial line and FiO_2 taken directly from mechanical ventilation settings.

[3] Patients randomized in the treatment group will receive Anakinra: SC treatment for 28 days (or until hospital discharge whichever comes first), alone or in combination with Tocilizumab: single IV injection on day 1 or Siltuximab: single IV injection on day 1. Some patients will only receive Tocilizumab: single IV injection on day1; Some patients will only receive Siltuximab: single IV injection on day 1.

[4] six-category ordinal scale (score measured daily up to hospital discharge, death, or the end of the study, whichever comes first):

1. Death
2. Hospitalized, on invasive mechanical ventilation or ECMO;
3. Hospitalized, on non-invasive ventilation or high flow oxygen devices;
4. Hospitalized, requiring supplemental oxygen
5. Hospitalized, not requiring supplemental oxygen
6. Not hospitalized

[5] Can be done at screening if C1D1 is on same day. Should be collected pre dose at D1.

Order of assessments:

IMP should always be administered after other assessments, where possible.

8.5. Restrictions for subjects during the study

There are no subject restrictions during this trial.

9. Sampling

9.1. Types and number of samples

D1: serum blood sample 2x 5ml, and optional EDTA blood sample, 4 x 10 ml
D6: serum blood sample 2x5ml, and optional EDTA blood sample, 4 x 10 ml
D15 or at discharge : serum blood sample 2x5ml, and optional EDTA blood sample, 4x10 ml

W10-20 follow-up visit :serum blood sample 2x5ml, optional EDTA blood sample, 4 x 10 ml

9.2. Timepoints of sampling

These samples are to be taken on D1, D6 and D15 and/or discharge and on final follow up visit between week 10 and 20. There's no time window allowed.

9.3. Sample Handling & Analysis

2 Serum samples (2x5 ml) will be taken during hospitalization together with the blood draw for standard of care on day 1, 6 and 15 or hospital discharge and safety follow up visit.
After clotting for 30-60 minutes the samples will be processed at 1500RPM or 410 g during 10 minutes at room temperature.
6 aliquots per time point will be filled and frozen at -80°C until further analysis.
Centrifugation and storage will be done by qualified personal at the various labs of the study centers.

Multiple cytokines (including IL-1 and IL-6 and chemokines will be measured by multiplex bead based ELISA assay.) This will be performed by a single central lab, at a time point decided by the coordinating principle investigator, after consulting with the other investigators. Samples will be shipped from the centers to the central lab, upon request of the coordinating principle investigator.

<u>OPTIONAL sampling per center</u>

In selected centers (to be decided by the local investigator after consulting with the coordinating principle investigator), additional 4x10 ml EDTA blood tubes will be taken at day 1, 6 and 15, or at hospital discharge and safety follow-up, for flow cytometric analysis and local research purposes. The investigators will purify peripheral blood mononuclear cells (PBMC) by gradient centrifugation. Results of these trial will be shared with the coordinating principle investigator after analysis.

Amongst other, we propose to explore potentially key immunological parameters before and weekly after initiation of experimental therapy to determine 1) their relationship with diseases severity and patient characteristics; 2) their modifications by experimental therapy; 3) their relationship with clinical outcome. The proposal is based on the use of systems biology to 1) explore immunological parameters that cannot be predicted by current knowledge of the immunopathology of the disease; 2) integrate large numbers of parameters in order to be able to identify the most strongly associated with clinical parameters and with the therapeutic intervention.

Flow cytometry will be performed on paraformaldehyde fixed samples. For UZ Gent, this will be done by Flanders Institute of Biotechnology (VIB), in one of their laboratories based at UZ Ghent.

Transcriptional program of immune cell populations: EDTA blood samples will be processed to purify peripheral blood mononuclear cells and stained for flow cytometric analysis of number of monocytes, HLA-DR expression on monocytes and dendritic cells, and lymphocyte activation.
Whole blood RNA sequencing will be performed to globally assess differences in transcriptional programing of immune cells between patients and modifications following therapy. These data will be used to identify most discordant patient groups and time points on which single cell RNA sequencing (CITE-Seq) will be conducted. On some samples at UZGent for example, a panel of 300 monoclonal antibodies coupled to oligonucleotides, developed by Martin Guilliams at VIB, will be used to identify and phenotype and immune cell populations that will be further analyzed for their transcriptional program, in collaboration with Ido Amit, Weizmann Institute.

In selected centres such as UZ Ghent, the plasma fraction of the EDTA blood tube after purification of the PBMC's will be used to measure SARS-CoV-2 RNA using quantitative q-RT-PCR. At UZ Ghent for example, this will be optimized by the virology lab of prof. Linos Vandekerckhove at UZ Gent and correlated if possible with viral load determined by nasopharyngeal swab detection on D1 and D6 (for patients simultaneously enrolled in the observational CO-VIM trial in UZ Ghent).

Aliquots of left-over serum will be used for a systems analysis of COVID-19 antibodies: Biophysical (subclasses, Fc glycosylation) and functional properties (macrophage and neutrophil phagocytosis, NK cell activation, complement activation, infection enhancement) of COVID-19 specific IgG and IgA will be assessed. A systems serology platform has been established at the Institute for Medical Immunology, ULB, in collaboration with Galit Alter, Rago Institute, and Margaret Ackerman, Dartmouth College.

9.4. Sample Storage and/or shipping

Serum samples and frozen PBMCs will be stored at FAGG-certified biobank facilities of the participating research centres.
Storage conditions: -80°C

9.5. Future use of stored samples

Initially samples will be stored for the use as described within this protocol. If at a later time point samples will be stored for future use, they will be stored in FAGG certified biobank.

10. Statistical Considerations

10.1. Sample size calculation

The study was powered to detect the two main effects of the 2x2 factorial design, assuming there is no effect modification (no interaction between the different treatments).
The first main effect relates to the comparison of IL6 blockers (SYLVANT® and Toclizumab groups combined) with usual care (2:1). To achieve at least 80% power to detect an improvement in median time to clinical improvement from 12 days to 8 days (corresponding to a hazard ratio of 1.5) at a two-sided significance level of 5%, assuming an allocation ratio of **1:2**, we need 215 events (i.e. clinical improvements of at least 2 points on the ordinal scale). With an accrual period of 8 weeks and a follow-up period for the last patient of 28 days, we would need 333 patients to observe 215 events, assuming 30% of patients are not susceptible to clinical improvement.
The second main effect relates to the comparison of the IL1 blocker (Anakinra) with usual care (1:2). To achieve at least 80% power to detect an improvement in median time to clinical improvement from 12 days to 8 days (corresponding to a hazard ratio of 1.5) at a two-sided significance level of 5%, assuming an allocation ratio of **2:1**, we need 215 events (i.e. clinical improvement of 2 points on the ordinal scale). With an accrual period of 8 weeks and a follow-up period for the last patient of 28 days, we would need **342 patients** to observe at least 215 events, assuming 30% of patients are not susceptible to clinical improvement.
The total number of patients needed to recruit within 8 weeks is 342 patients.
Sample size calculations were performed using R version 3.6.1 (2019-07-05) with the nSurvival function from the “gsDesign” package.

10.2. Type of statistical methods

Primary analysis

For each of the two main comparisons, the primary analysis is based on the primary endpoint defined as time from randomisation until either an improvement of two points on the six-category ordinal scale (defined above) or discharge from the hospital. Patients who died before having experienced a 2-point improvement will be censored at the longest observed follow-up time seen in the study. If a patient dies after having had clinical improvement, we will consider this patient as a patient who reached the event of clinical improvement.

Kaplan-Meier estimates of improvement-free survival curves will be compared between treatment groups with the log-rank test. The cumulative improvement rate will be plotted as a function of observation time.
In addition, stratified Cox proportional hazards regression models for time to clinical improvement (expressed in days) will be fitted with treatment group (IL6-blocking treatment versus usual care OR Anakrina versus usual care) as fixed effect. All models will be stratified by center, to allow for a different baseline hazard for clinical improvement at each center. The hazards ratio with 95% confidence interval will be estimated from this model. The proportional hazards assumption for the treatment effect will be checked by visual inspection of the log-log survival curves estimated non-parametrically.
Primary analysis will be according to the intention-to-treat principle, including all patients randomized and where patients allocated to a treatment group will be analyzed as members of that group irrespective of their compliance to the planned course of treatment.
All analyses will be performed using R software. The survival analyses will be done using the “survival” package.

Given the short duration of the study (8 weeks for recruitment + 28 days for follow-up of the last patient), no interim analyses are planned.

10.3. Statistical analysis team

Biostatistics Unit, Faculty of Medicine and Health Science, Ghent University

11. Data handling

11.1. Method of data collection

Subjects that are included in the study, will be assigned a unique study number upon their registration in REDCap. On all documents submitted to the coordinating center, sponsor or CI, patients will only be identified by their study number. The subject identification list will be safeguarded by the site. The name and any other directly identifying details will not be included in the study database.

11.1.1. Case Report Form

An electronic data capture (EDC) system, i.e. REDCap, will be used for data collection. Data reported on each eCRF should be consistent with the source data. If information is not known, this must be clearly indicated on the eCRF. All missing and ambiguous data will be clarified.
Only the data required by the protocol are captured in the eCRF. The eCRFs and the database will be developed, based on the protocol. The final eCRF design will be approved by the Co-ordinating Investigator.
All data entries and corrections will only be performed by study site staff, authorized by the investigator. Data will be checked by trained personnel (monitor, data manager) and any errors or inconsistencies will be clarified. The investigator must verify that all data entries in the eCRF are accurate and correct.

REDCap is provided and maintained by Vanderbilt University; a license for use was granted to the Health, Innovation and Research Institute (HIRUZ). REDCap is a web-based system.
The study site staff is responsible for data entry in REDCap.

11.1.2. Data directly collected in the CRF (no source available)

N.A.

11.2. Data storage

The data is accessed through a web browser directly on the secure REDCap server. The server is hosted within the UZ Ghent campus and meets hospital level security and back-up requirements.

Privacy and data integrity between the user's browser and the server is provided by mandatory use of Transport Layer Security (TLS), and a server certificate issued by TERENA (Trans-European Research and Education Networking Association). All study sites will have access to REDCap. Site access is controlled with IP restriction.

11.3. Archiving of data

The investigator and sponsor specific essential documents will be retained for at least 25 years. At that moment, it will be judged whether it is necessary to retain them for a longer period, according to applicable regulatory or other requirement(s).

11.4. Access to data

Direct access will be granted to authorised representatives from the Sponsor, host institution and the regulatory authorities to permit study-related monitoring, audits and inspections.

Login in REDCap is password controlled. Each user will receive a personal login name and password and will have a specific role which has predefined restrictions on what is allowed in REDCap. Furthermore, users will only be able to see data of subjects of their own site. Any activity in the software is traced and transparent via the audit trail and log files.

Study Data, Data Ownership and Data Sharing with KCE

After the completion of the study the Sponsor will transfer the pseudonymized study data set to KCE. KCE will request approval from the competent chamber of the Information Security Committee to have the relevant study data linked with IMA data by a trusted third party (TTP, eHealth platform) using the patient national number.

The patient information and consent includes wording that the **national number** will be recorded on site by the investigator for later data linkage. The patient information and consent will also include that in case the patient is randomized, it is planned that a trusted third party (TTP, eHealth platform) will receive and use the national number to link with IMA administrative data. This data linkage is planned to obtain a more complete data set that will be used for the analysis of effectiveness and cost-effectiveness of the intervention by KCE.

KCE and Sponsor have entered into a research agreement detailing the roles and responsibilities of each party, as well as other legal aspects of this collaboration, including the right to use and access of KCE to the Study Data.

“Background” means any intellectual property (IP), data, materials, information owned or controlled by the Sponsor or a Site, and required to run this Study. Sponsor will identify such Background including the legal restrictions of which Sponsor or Sites are aware that may affect the use of the Background for the purpose of the Study or the rights granted to KCE under this Agreement.

The Study Data consist of this protocol, including amendments, the electronic forms for data capture, including the annotations and guidance for use, the electronic database of the pseudonymized clinical and non-clinical data collected using data capture, including the log of changes from data entry to database lock, study reports based on these pseudonymized data, and any data or reports generated at a later stage, eg based on exploratory analyses or stored samples.

“Foreground” means any Study Data, and any tangible biological, chemical and physical material and inventions, that are generated, acquired, discovered, conceived, developed, created, exemplified or derived as a result of carrying out the Clinical Study, whatever its form or nature, whether it can be protected or not, as well as any Foreground IP. Sponsor acknowledges that the main purpose of the research performed under this Agreement is to generate results that will serve the general public interests, and specifically the interests of the patients and public healthcare decision making bodies,

and, therefore, undertakes not to exploit the Foreground in any way that is or could be detrimental to such interests.

The Sponsor owns the Study Data, but provides KCE with a copy of the pseudonymized database after database lock as well as a royalty-free unrestricted license to use the Study Data for non-commercial public health related purposes as detailed in the Agreement between KCE and UZ Gent. If judged appropriate, KCE will introduce the request to the competent chamber of the Information Security Committee and arrange for the data linkage. For the sake of clarity, the linked data are not part of the Study Data. However, KCE will discuss with the Sponsor the results of the analyses and the reporting of the linked data.

12. Safety

12.1. Definitions

Term	Definition
**Adverse Event (AE)**	Any untoward medical occurrence in a subject to whom a medicinal product has been administered, including occurrences which are not necessarily caused by or related to that product.
**Unexpected Adverse Event**	An adverse event, the nature or severity of which is not consistent with the applicable product information (e.g., Investigator's Brochure for an unapproved investigational product or package insert/summary of product characteristics for an approved product).
**Adverse Reaction (AR)**	An untoward and unintended response in a subject to an investigational medicinal product which is related to any dose administered to that subject.<br>The phrase "response to an investigational medicinal product" means that a causal relationship between a study medication and an AE is at least a reasonable possibility, i.e. the relationship cannot be ruled out.<br>All cases judged by either the reporting medically qualified professional or the Sponsor as having a reasonable suspected causal relationship to the study medication qualify as adverse reactions.
**Serious Adverse Event (SAE)**	A serious adverse event is any untoward medical occurrence that:<br>• results in death<br>• is life-threatening<br>• requires inpatient hospitalisation or prolongation of existing hospitalisation<br>• results in persistent or significant disability/incapacity<br>• consists of a congenital anomaly or birth defect<br>Other ‘important medical events’ may also be considered serious if they jeopardise the subject or require an intervention to prevent one of the above consequences.<br>NOTE: The term "life-threatening" in the definition of "serious" refers to an event in which the subject was at risk of death at the time of the event; it does not refer to an event which hypothetically might have caused death if it were more severe.
**Serious Adverse Reaction (SAR)**	An adverse event that is both serious and, in the opinion of the reporting Investigator, believed with reasonable probability to be due to one of the study treatments, based on the information provided.
**Suspected Unexpected Serious Adverse Reaction (SUSAR)**	A serious adverse reaction, the nature and severity of which is not consistent with the information about the medicinal product in question set out:<br>• in the case of a product with a marketing authorisation, in the summary of product characteristics (SmPC) for that product<br>• in the case of any other investigational medicinal product, in the investigator’s brochure (IB) relating to the study in question

<u>Attribution definitions</u>
An adverse event is considered associated with the use of the drug if the attribution is possible, probable or definitive.

Not related
An adverse event which is not related to the use of the drug.

Unlikely
An adverse event for which an alternative explanation is more likely - e.g. concomitant drug(s), concomitant disease(s), and/or the relationship in time suggests that a causal relationship is unlikely.

Possible
An adverse event which might be due to the use of the drug. An alternative explanation - e.g. concomitant drug(s), concomitant disease(s), - is inconclusive. The relationship in time is reasonable; therefore the causal relationship cannot be excluded.

Probable
An adverse event which might be due to the use of the drug. The relationship in time is suggestive (e.g. confirmed by dechallenge). An alternative explanation is less likely - e.g. concomitant drug(s), concomitant disease(s).

Definitely
An adverse event which is listed as a possible adverse reaction and cannot be reasonably explained by an alternative explanation - e.g. concomitant drug(s), concomitant disease(s). The relationship in time is very suggestive (e.g. it is confirmed by dechallenge and rechallenge).

12.2. Reporting requirements

12.2.1. AE reporting

AE’s will be recorded from randomisation until the end of the study, as defined in section 4.2.
Special attention will be given to those subjects who have discontinued the study for an AE, or who experienced a severe or a serious AE. All AE’s should be recorded in the patient’s file and in the CRF.

12.2.2. SAE reporting

SAE’s occurring during the entire study period will be reported as below.

All serious adverse events (initial and follow up information) and pregnancies occurring during this study must be reported by the local Principal Investigator within 24 hours after becoming aware of the SAE to:

- The local ethics committee (it is the responsibility of the local PI to report the local SAE’s to the local EC)
- HIRUZ CTU of the University Hospital Ghent
- The National Coordinating Investigator (in case of multicenter studies)

This reporting is done by using the appropriate SAE form. For the contact details, see below.

12.2.3. SUSAR reporting

In case the Coordinating Investigator, in consultation with HIRUZ CTU, decides the SAE is a SUSAR (Suspected Unexpected Serious Adverse Reaction), HIRUZ CTU will report the SUSAR to the Central EC

and the FAMHP within the timelines as defined in national legislation. The Coordinating Investigator reports the SUSAR to all local PI's.
In case of a life-threatening and fatal SUSAR the entire reporting process must be completed within 7 calendar days. In case of a non life-threatening SUSAR the reporting process must be completed within 15 calendar days.

12.3. List of contact details for safety reporting

HIRUZ CTU:
Ghent University Hospital
C. Heymanslaan 10, 1K5
9000 Ghent, Belgium
e-mail: hiruz.ctu@uzgent.be
Tel: +32 9 332 05 00
Fax: +32 9 332 05 20

Coordinating Investigator:
Prof. dr. Bart Lambrecht
Ghent University Hospital
Department of pneumology
C. Heymanslaan 10, 1K5
9000 Ghent, Belgium
email: bart.lambrecht@ugent.be
Tel: +32 9 332 91 10

Marketing Authorisation Holder:
Various companies

SOBI
ROCHE
EUSAPHARMA

12.4. Flowchart Reporting

Type of Adverse Event	*Action to be taken*
AE	List all AE's per subject in the patient's file and add this information to the CRF.
SAE	Notify to HIRUZ CTU within 24 hours after becoming aware of the SAE + add the SAE to a list that will be reported yearly (see section 13.8)
SAR	Notify to HIRUZ CTU within 24 hours after becoming aware of the SAE<br>→ HIRUZ CTU will submit to the central EC<br>→ study team informs company that provides the IMP
SUSAR	Notify to HIRUZ CTU within 24 hours after becoming aware of the SUSAR

	→ HIRUZ CTU will submit to the central EC.<br>→ HIRUZ CTU will submit to the FAMHP<br>→ study team informs company that provides the IMP

In case the (SU)SAR occurs at a local participating site, the local PI or study team should also contact:

- The local Ethics Committee
- The Co-ordinating Investigator

12.5. Events, excluded from reporting

COVID-19 infection is a very recent syndrome, on which few data are available. Normal symptoms and natural disease course symptoms that will not be reported as adverse events are dyspnea, coughing, malaise, fever, drop in oxygen saturation, progression to respiratory failure, progression to ARDS, drop in blood pressure, progression to multi-organ failure.

12.6. Data Safety Monitoring Board (DSMB)

All study medication is registered and used in current practice. Despite the known safety profile of the study medications and study design, a DSMB is foreseen.

12.7. Development Safety Update Report

The Coordinating Investigator will provide DSURs once a year throughout the clinical study, or on request, to the Competent Authority (FAMHP in Belgium), Ethics Committee and Sponsor. This DSUR will include all SAE's (who were not categorized as SAR's and were not immediately reported to the EC).

The report will be submitted within 60 days after the start of the study, and will subsequently be submitted each year until the study is declared ended.

HIRUZ CTU can provide a template that can be used to complete this DSUR.

13. Monitoring/Auditing/Inspection

13.1. Monitoring

13.1.1. General

Monitoring of the study will be performed in compliance with GCP E6(R2) and the applicable regulatory requirements. The study team will be trained in an initiation visit by the monitor. A training and delegation log will be held. A detailed description of the monitoring tasks can be found in the latest version of the (study-specific) 'Monitoring plan'.

13.1.2. Monitoring team

Monitoring services will be provided by HIRUZ CTU. All relevant contact details (e.g. primary contact person, can be found in the 'Monitoring plan'.

13.1.3. Scope

Monitoring services will consist of the following (non-exhaustive list):
- review of informed consents and the followed process
- check on recruitment status
- checking for protocol deviations/violations
- checking GCP compatibility
- check on safety reporting compliance
- IMP handling and storage
- review of study data

…

13.2. Inspection

This study can be inspected at any time by regulatory agencies during or after completion of the study. Therefore access to all study records, including source documents, must be accessible to the inspection representatives. Subject privacy must be respected at all times, in accordance to GDPR, GCP and all other applicable local regulations.
The investigator/study team should immediately notify the sponsor if he or she has been contacted by a regulatory agency concerning an upcoming inspection.

13.3. Protocol Deviation policy

Sponsor and all investigators agree to take any reasonable actions to correct protocol deviations/violations noted during monitoring/inspection, in consultation with the monitoring team. All deviations must be documented on a protocol deviation log by the study team that is kept available at any time for monitoring/inspection purposes. Under emergency circumstances, deviations from the protocol to protect the rights, safety or well-being of human subjects may proceed without prior approval of the sponsor and the EC.

13.4. Serious breach to GCP and/or the protocol

Critical issues that significantly affect patient safety, data integrity and/or study conduct should be clearly documented and will be communicated with the Coordinating Investigator, HIRUZ CTU and possibly both the applicable Ethics Committee(s) and Competent authority. (Please contact HIRUZ CTU asap in case of a serious breach: hiruz.ctu@uzgent.be and/or +3293320500).
Early determination of the study (in a specific center or overall) may be necessary in case of major non-compliance.

14. Ethical and legal aspects

14.1. Good Clinical Practice

The study will be conducted cfr the latest version of the ICH E6 (R2) GCP guidelines, creating a standard for the design, conduct, performance, monitoring, auditing, recording, analyses and reporting of clinical studies that provides assurance that the data and reported results are accurate and that the rights, integrity and confidentiality of study subjects are protected.

14.2. Informed Consent

Eligible subjects may only be included in the study after providing written (witnessed, if needed) Ethics Committee-approved informed consent, or, if incapable of doing so, after such consent has been provided by a legally acceptable representative(s) of the subject.
Informed consent must be obtained before conducting any study-specific procedures (as described in this protocol).

Prior to entry in the study, the investigator must explain to potential subjects or their legal representatives the study and the implication of participation. Subjects will be informed that their participation is voluntary and that they may withdraw consent to participate at any time. Participating subjects will be told that their records may be accessed by competent authorities and by authorized persons without violating the confidentiality of the subject, to the extent permitted by the applicable law(s) and/or regulations. By signing the Informed Consent Form (ICF), the subjects or legally acceptable representatives are authorizing such access.
After this explanation and before entry to the study, written, dated and signed informed consent should be obtained from the subject or legally acceptable representative. The ICF should be provided in a language sufficiently understood by the subject. Subjects must be given the opportunity to ask questions.
The subject or legally acceptable representative will be given sufficient time to read the ICF and to ask additional questions. After this explanation and before entry to the study, consent should be appropriately recorded by means of either the subject's or his/her legal representative's dated signature or the signature of an independent witness who certifies the subject's consent in writing. After having obtained the consent, a copy of the ICF must be given to the subject.
In case the subject or legally acceptable representative is unable to read, an impartial witness must attest the informed consent.
Subjects who are unable to comprehend the information provided or pediatric subjects can only be enrolled after consent of a legally acceptable representative.
The following information should be added to the electronic patient dossier (EPD):

- which version of the ICF was obtained
- who signed the ICF
- if sufficient time has been given to consider participation into the study
- which investigator obtained ICF with the date of signature
- if a copy was provided to the patient
- start and end of participation in the study

14.3. Approval of the study protocol

14.3.1. General

The protocol has been reviewed and approved by the Ethics Committee of the Ghent University (Hospital), designated as the central Ethics Committee, after consultation with the local Ethics Committees, and the Federal Agency for Medicine and Health Products (FAMHP). This study cannot start before both approvals have been obtained.

14.3.2. Protocol amendments

Any significant change or addition to the protocol can only be made in a written protocol amendment that must be approved by the Central Ethics Committee (and the FAMHP if applicable).
Only amendments that are intended to eliminate an apparent immediate safety threat to patients may be implemented immediately.
Notwithstanding the need for approval of formal protocol amendments, the investigators are expected to take any immediate action, required for the safety of any subject included in this study, even if this action represents a deviation from the protocol. These actions should always be notified to the sponsor.

14.4. Confidentiality and Data Protection

All study data will be handled in accordance with the law on General Data Protection Regulation (GDPR) and institutional rules
[Belgian law dated on 30 July 2018 and 22 Aug. 2002].

The collection and processing of personal data from subjects enrolled in this study will be limited to those data that are necessary to fulfil the objectives of the study. These data must be collected and processed with adequate precautions to ensure confidentiality and compliance with applicable data privacy protection laws and regulations.

Appropriate technical and organizational measures to protect the personal data against unauthorized disclosures or access, accidental or unlawful destruction, or accidental loss or alteration must be put in place. Sponsor and site personnel whose responsibilities require access to personal data agree to keep the identity of subjects confidential.
The informed consent obtained from the subject includes explicit consent for the processing of personal data and for the investigator/institution to allow direct access to his or her original medical records (source data/documents) for study-related monitoring, audit, Ethics Committee review and regulatory inspection. This consent also addresses the transfer of the data to other entities, if applicable.

Privacy and confidentiality of data generated in the future on stored samples will be protected by the same standards applicable to all other clinical data.
Stored samples will be pseudonymized throughout the sample storage and analysis process and will not be labelled with personal identifiers.

14.5. Liability and Insurance

The sponsor has taken a no fault insurance for this study, in accordance with the relevant legislation (article 29, Belgian Law of May 7, 2004).
Sponsor: Ghent University Hospital
Insurance Details: Allianz Global Corporate & Specialty; Uitbreidingstraat 86, 2600 Berchem; Tel: +32 33 04 16 00
Polis number: BEL000862

14.6. End of Study Notification

If all subjects have completed the study, a notification of the end of the study should be submitted to the (Central) Ethics Committee and FAMHP. This notification should be made within 90 days of the end of the clinical study. In case of early termination (definition in CT-1, 4.2), this is reduced to 15 days.

15. Publication policy

This study will be registered at ClinicalStudies.gov, and results information from this study will be submitted to ClinicalStudies.gov. In addition, every attempt will be made to publish results in peer-reviewed journals.

16. Reference List

1. C. Wu *et al.*, Risk Factors Associated With Acute Respiratory Distress Syndrome and Death in Patients With Coronavirus Disease 2019 Pneumonia in Wuhan, China. *JAMA Intern Med*, (2020).
2. C. Huang *et al.*, Clinical features of patients infected with 2019 novel coronavirus in Wuhan, China. *Lancet* **395**, 497-506 (2020).
3. B. Shakoory *et al.*, Interleukin-1 Receptor Blockade Is Associated With Reduced Mortality in Sepsis Patients With Features of Macrophage Activation Syndrome: Reanalysis of a Prior Phase III Trial. *Crit Care Med* **44**, 275-281 (2016).
4. F. van Rhee *et al.*, Siltuximab for multicentric Castleman's disease: a randomised, double-blind, placebo-controlled trial. *Lancet Oncol* **15**, 966-974 (2014).
5. P. Mehta *et al.*, COVID-19: consider cytokine storm syndromes and immunosuppression. *Lancet* **395**, 1033-1034 (2020).
6. W. Zhang *et al.*, The use of anti-inflammatory drugs in the treatment of people with severe coronavirus disease 2019 (COVID-19): The experience of clinical immunologists from China. *Clin Immunol* **214**, 108393 (2020).
7. D. Wang *et al.*, Clinical Characteristics of 138 Hospitalized Patients With 2019 Novel Coronavirus-Infected Pneumonia in Wuhan, China. *JAMA*, (2020).
8. F. Bennardo, C. Buffone, A. Giudice, New therapeutic opportunities for COVID-19 patients with Tocilizumab: Possible correlation of interleukin-6 receptor inhibitors with osteonecrosis of the jaws. *Oral Oncol*, 104659 (2020).
9. W. H. Organization, WHO R&D Blueprint Informal consultation on teh potential role of IL-6/IL-1 antagonists in teh clinical management of COVID-19 infection. *WAO Website* https://www.who.int/blueprint/priority-diseases/key-action/novel-coronavirus/en/, (2020).
10. D. Wu, X. O. Yang, TH17 responses in cytokine storm of COVID-19: An emerging target of JAK2 inhibitor Fedratinib. *J Microbiol Immunol Infect*, (2020).
11. Z. Xu *et al.*, Pathological findings of COVID-19 associated with acute respiratory distress syndrome. *Lancet Respir Med*, (2020).
